# Automatic delineation and quantification of pulmonary vascular obstruction index in patients with pulmonary embolism using Perfusion SPECT-CT: a simulation study

**DOI:** 10.1186/s40658-021-00396-1

**Published:** 2021-07-05

**Authors:** David Bourhis, Laura Wagner, Julien Rioult, Philippe Robin, Romain Le Pennec, Cécile Tromeur, Pierre Yves Salaün, Pierre Yves Le Roux

**Affiliations:** 1 Service de Médecine Nucléaire, Centre Hospitalier Régional Universitaire de Brest, Brest, France; 2 EA3878 GETBO, Université de Bretagne Occidentale, Brest, France; 3 Service de Pneumologie, Centre Hospitalier Régional Universitaire de Brest, Brest, France

**Keywords:** V/Q SPECT-CT, Lung function, PE, Simulation, Segmentation

## Abstract

**Background:**

In patients with pulmonary embolism (PE), there is a growing interest in quantifying the pulmonary vascular obtruction index (PVOI), which may be an independent risk factor for PE recurrence. Perfusion SPECT/CT is a very attractive tool to provide an accurate quantification of the PVOI. However, there is currently no reliable method to automatically delineate and quantify it. The aim of this phantom study was to assess and compare 3 segmentation methods for PVOI quantification with perfusion SPECT/CT imaging.

**Methods:**

Three hundred ninety-six SPECT/CT scans, with various PE scenarios (*n* = 44), anterior to posterior perfusion gradients (*n* = 3), and lung volumes (*n* = 3) were simulated using Simind software. Three segmentation methods were assesssed: (1) using an intensity threshold expressed as a percentage of the maximal voxel value (MaxTh), (2) using a Z-score threshold (ZTh) after building a Z-score parametric lung map, and (3) using a relative difference threshold (RelDiffTh) after building a relative difference parametric map. Ninety randomly selected simulations were used to define the optimal threshold, and 306 simulations were used for the complete analysis. Spacial correlation between PE volumes from the phantom data and the delineated PE volumes was assessed by computing DICE_PE_ indices. Bland-Altman statistics were used to calculate agreement for PVOI between the phantom data and the segmentation methods.

**Results:**

Mean DICE_PE_ index was higher with the RelDiffTh method (0.85 ± 0.08), as compared with the MaxTh method (0.78 ± 0.16) and the ZTh method (0.67 ± 0.15). Using the RelDiffTh method, mean DICE_PE_ index remained high (> 0.81) regardless of the perfusion gradient and the lung volumes. Using the RelDiffTh method, mean relative difference in PVOI was − 12%, and the limits of agreement were − 40% to 16%. Values were 3% (− 75% to 81%) for MaxTh method and 0% (− 120% to 120%) for ZTh method. Graphycal analysis of the Bland-Altman graph for the RelDiffTh method showed very close estimation of the PVOI for small and medium PE, and a trend toward an underestimation of large PE.

**Conclusion:**

In this phantom study, a delineation method based on a relative difference parametric map provided a good estimation of the PVOI, regardless of the extent of PE, the intensity of the anterior to posterior gradient, and the whole lung volumes.

**Supplementary Information:**

The online version contains supplementary material available at 10.1186/s40658-021-00396-1.

## Background

Lung ventilation/perfusion (V/Q) scintigraphy is a well established test for pulmonary embolism (PE) diagnosis [1, 2]. V/Q planar scan has been validated in large management oucome studies [3–5]. Development of imaging equipment and radiopharmaceuticals has then allowed the introduction of V/Q single photon emission computed tomography (SPECT) scintigraphy, and more recently V/Q SPECT/CT, which has been reported to improve the diagnostic performance of the test [6, 7] and has been widely adopted in daily practice [8]. Besides the diagnosis of PE itself, there has been a growing interest in quantifying the extent of vascular obstruction of PE. Indeed, several studies reported that the pulmonary vascular obstruction index (PVOI) measured on lung perfusion scintigraphy, either at diagnosis or at completion of anticoagulant therapy, may be an independent risk factor for PE recurrence and chronic thromboebolic pulmonary hypertension (CTEPH) [9, 10]. PVOI quantification by planar imaging relies on the application of the Meyer score [11], which was validated on perfusion imaging using pulmonary angiography as a reference standard, but which is complex to use in daily practice and involves a certain amount of subjectivity. By using 3-dimensional imaging, lung SPECT has an inherent technical advantage over conventional 2-dimensional planar imaging through its ability to eliminate overlap of activities, its visualization of the medial-basal segment, and its ability to better characterize the size, shape, and location of defects [12]. Perfusion SPECT/CT is therefore a very attractive tool to improve the accuracy and reproducibility of PVOI quantification. However, there is currently no reliable method to delineate and quantify the PVOI with Perfusion SPECT/CT imaging. Indeed, the delineation of lung perfusion is complex because of the physiological non-uniformity of lung perfusion, with especially an anterior-to-posterior intensity gradient of variable intensity across patients. In that respect, similarly to a method used for brain imaging (Statistical Parametric Mapping (SPM) [13]), a method based on the co-registration with physiological images and a voxelwize analysis may be of value. Another issue for the assessment and validation of segmentation methods of lung perfusion with perfusion SPECT/CT is the lack of ground truth. In order to overcome these problems, Monte-Carlo simulations may be an intersting tool. First, it would solve the problem of the reference standard. Second, it would allow to test a large number of segmentation methods and clinical scerarios. Finally, simulations may integrate confounding factors, such as the impact of gravity on the macroaggregated albumin distribution, that make conventional delineation methods inacurate in some situations. Our group recently generated 3D mean and standard deviation statistical maps of regional lung function based on free-form co-registration of 73 normal V/Q SPECT/CT scans, with the aim of testing original methods for lung function delineation based on an a-priori knowledge of normality [14]. In this work, we found that the main factor of variability across patients with a normal perfusion SPECT/CT was the intensity of the anterior to posterior gradient. In a second work, we therefore developed and validated a dual isotopes lung V/Q SPECT-CT model for Monte Carlo studies, integrating the anterior-to-posterior gradient on perfusion images [15].

The aim of this phantom study was to test and compare 3 different delineation methods to automatically compute the PVOI on perfusion SPECT/CT imaging: a conventional method using the same intensity threshold for all pixels, and 2 original methods based on the coregistration and comparison with physiological statistical maps, and a voxelwise analysis (Z-score threshold and relative difference threshold methods).

## Materials and methods

### Overall methology for Perfusion SPECT/CT simulations

In order to test the various delineation methods, we simulated a large spectrum of PE, with various size and location (44 clinical scenarios), on lung perfusion SPECT/CT. Three different sizes of lungs were simulated in order to assess the impact of the statistical maps’ deformable registrations. As the variability of the intensity of the anterior to posterior gradient is a key issue for the delineation of lung perfusion volumes, we also simulated three different anterior to posterior intensity gradients. Overall, 44 × 3 × 3 = 396 perfusion SPECT/CT scans were therefore simulated. As PVOI is a quantification of lung perfusion, ventilation delineation was not assessed in this work, but it was integrated in the model to improve the realism of simulations in terms of Compton scattering.

### PE models definition

To define a catalog of PE, CT examinations of real patients were delineated. A senior nuclear medicine physician with experience in reading V/Q scan delineated the lobes following the fissures and the lung segments based on the bronchial tree. The segments were then divided to obtain two half segments. We defined 44 models of PE, selecting the lobes, segments, and sub-segments to be turned off for simulations. We defined 8 sub-segmental PE (one or multiple sub-segments), 10 single segmental PE, 19 multi segmental PE (from 2 to 14 segments), 5 lobar PE, and 2 multi lobar PE. The exact definition of the models can be found in the supplementary material.

### Lung SPECT simulations and reconstructions

All simulations were run with Simind software [16]. Realistic dual isotopes lung V/Q SPECT scans were simulated using a methodology described in a previous work [15]. Briefly, we used CT data from real SPECT-CT examinations acquired on a Symbia T6 system (Siemens, Erlangen) equipped with a medium energy low penetration (MELP) collimator. The camera modeling parameters were set in order to correspond to this system as it is used for dual isotopes V/Q SPECT-CT [17]. CT data (low-dose free breathing CT) were used to define three simulation digital phantoms corresponding to small (1915 mL), regular (2730 mL), and large (3515 mL) lungs. These scans were selected so that the lung volume fit the mean, the mean plus one, and mean minus one standard deviation volume, measured on the database of 73 normal cases [14]. Simulation geometries were Zubal-like phantoms [18, 19], built from the CT. CT data were segmented according to hounsfield units using MiM software (7.0, Cleveland). Six representative tissues were delineated, including outside air, bones, fat, soft tissues, lungs, bronchi, and the PE area. A code was assigned to each area. Images bit depth was set to 8 bits, and a unique value was attributed to each segmented area using ImageJ sofware [20]. Those values were used in Simind to set the desired value of density and radioactivity concentration in the defined areas. Digital phantom was sub-sampled in a 128^2^ matrix to accelerate the simulation calculation, and the simulation grid was 128 × 128 × 108 matrix, corresponding to 3.92 × 3.92 × 3.59 mm voxels. With regard to ventilation, the simulated radioactivity was evenly set to 55 kBq.mL^− 1^ in the lungs and the airways. With regard to perfusion, in order to model the anterior to posterior gradient, lungs were divided into sixteen coronal planes. For each coronal plane, a relative to maximum radioactivity concentration value was assigned. Three different sets of values were defined to model a weak, regular, and a strong gradient. The gradients were defined to fit the mean, the mean plus one, and mean minus one standard deviation anterior to posterior intensity gradient measured on the database of 73 normal cases [14]. Radioactivity concentrations rose from the first to the last coronal plane to define weak, regular, and strong gradients (64 to 85 kB.mL^− 1^, 49 to 98 kB.mL^− 1^ and 29 to 116 kB.mL^− 1^) for the small phantom, 52 to 67 kB.mL^− 1^, 42 to 78 kB.mL^− 1^ and 27 to 95 kB.mL^− 1^ for the regular phantom, and 44 to 56 kB.mL^− 1^, 36 to 67 kB.mL^− 1^, and 24 to 85 kB.mL^− 1^ for the large phantom. As the source map is different when simulating ventilation and perfusion, simulations were not run simultaneously. Photons emitted from ^99m^Tc decay were simulated with a 140 keV energy and 88.5% abundance. As Krypton gas is continuously inspired and expired and has a very fast decay (half-life is 13 s), it was simulated as a stationary gas without significant decay, with homogeneous concentration, with a 190 keV energy and 100% abundance. Scatter data was stored at each energy window, and ^81m^Kr scatter was added to ^99m^Tc lower scatter and primary energy windows. All SPECT reconstructions were performed on Siemens MI-Apps software , with FLASH3D, 4 iterations, 8 subsets, and 8.4 mm gaussian post filtering, scatter correction (double energy windows method), with attenuation correction. Overall, we defined 44 models of PE, simulated on three different geometries (small, regular, and large lungs) with three different radioactivity source maps (weak, regular, and strong anterior to posterior intensity gradient). Three hundred ninety-six datasets were therefore reconstructed.

### Segmentation methods

We evaluated three segmentation methods of lung volumes with normal perfusion. First, we tested a fixed intensity threshold for all pixels, expressed as a percentage of the maximal pixel value (MaxTh), with six different thresholds (10%, 15%, 20%, 25%, 30%, 35%), applied on both lungs. Then, we tested two original methods, based on the quantification of abnormality, inspired by the SPM method used for brain imaging: a Z-score map threshold method (ZTh), and a relative difference map threshold (RelDiffTh) method. These methods were applied using mean (NMmap) and a standard deviation (NSDmap) maps built in a previous study from 73 normal V/Q SPECT/CT cases [14]. For each simulation dataset, we performed a free-form registration, based on CT data, of the NMmap and NSDmap up to the simulated SPECT-CT. Then, we normalized the simulated SPECT to the mean intenity value, and we computed two parametric maps, a Z-score map (Zmap) and a relative difference map (RelDiffmap) as follows:

$$ {Zmap}_{simulated\ SPECT}\left(x,y,z\right)=\frac{\left[ normSPECTPixelvalue\left(x,y,z\right)- NMmapValue\left(x,y,z\right)\right]}{NSDmapValue\left(x,y,z\right)}; $$

$$ {RelDiffmap}_{simulated\ SPECT}\left(x,y,z\right)=\frac{\left[ normSPECTPixelvalue\left(x,y,z\right)- NMmapValue\left(x,y,z\right)\right]}{NMmapValue\left(x,y,z\right)}; $$

**Fig. 1:**
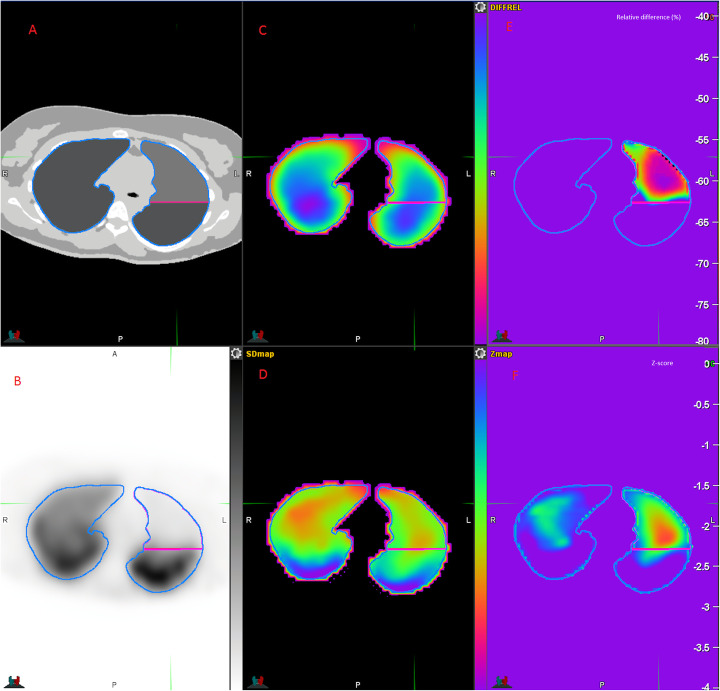
Example of simulated PE with corresponding parametric maps: digital phantom (**a**), simulated SPECT (**b**), registered NMmap (**c**), and NSDmap (**d**), corresponding calculated RelDiffmap (**e**) and Zmap (**f**). The Zmap shows a defect in the right upper lobe while there is no perfusion defect. The RelDiffMap provides a better contrast between functional and no functional areas

An example of the different datasets of one case is shown in Fig. 1. Finally, we tested several thresholds on Zmap (− 0.6, − 0.8, − 1, − 1.2, − 1.4, − 1.6) and RelDiffmap (− 30%, − 40%, − 50%, − 60%).

As the threshold tool delineated pixels whose intensities were higher than the threshold values, only the functional lung volumes where segmented for the three methods.

### Quantitative measurements

The whole lung volume (WLv) was the volume computed from CT data. From the phantom data, the ground truth volumes were computed, including the functional lungs volume (FLv_ph_) and the PE volume (PEv_ph_). Ground truth PVOI was calculated as follows : PVOI_ph_ = PEv_ph_/WLv. For each simulation, segmentation method and threshold value, the segmented functional lungs (FLv_s_) were stored and the PE volume (PEv_s_) and simulation PVOI were calculated as follows: PEv_s_ = WLv-FLv_s_, PVOI_s_ = PEv_s_/WLv. To measure the efficiency of the segmentation, DICE indices (DICE_PE_) of segmented PE volumes (PEv_s_) and phantom PE volumes (PEv_ph_) were calculated as follows DICE_PE_ = (2xPEv_s_∩PEv_ph_)/( PEv_s_ + PEv_ph_).

### Statistical analysis

All measurements were separated in two batches. 10 models of PE (90 simulations) were randomly selected to define the best threshold for each segmentation method. The best threshold was defined as the threshod which provided the highest mean DICE_PE_ index for each method. Thirty-four models (306 simulations) were available for the complete analysis. DICE_PE_ were expressed graphically as [min;Q1;median;mean;Q3;max] and quantitatively as mean (± SD). DICE PE indexes distributions were compared using a Wilcoxon signed rank test. A p value < 0.05 was considered statistically significant. The correlations between PVOI_ph_ and PVOI_s_ were tested using Pearson’s r correlation coefficient, and a Bland and Altman visual analysis was performed in terms of absolute difference and relative difference.

## Results

### Calibration

**Fig. 2:**
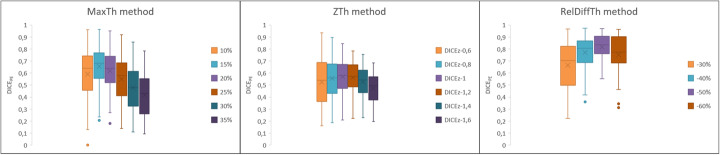
Determination of the best threshold for the 3 segmentation methods. DICE_PE_ coefficients were calculated on 90 randomly selected models using various thresholds. The best thresholds were 15% for the MaxTh method, Z = − 1 for the ZTh method and − 50% for the RelDiffTh method

Using the MaxTh method, mean (± SD) DICE_PE_ coefficients were 0.59 (0.23), 0.66 (0.18), 0.62 (0.18), 0.55 (0.19), 0.48 (0.19), and 0.2 (0.18) with the 10%, 15%, 20%, 25%, 30%, and 35% thresholds, respectiveley. Using the ZTh method, mean (± SD) DICE_PE_ coefficients were 0.53 (0.2), 0.56 (0.18), 0.57 (0.15), 0.56 (0.13), 0.53 (0.13), and 0.47 (0.12) with the − 0.6, − 0.8, − 1, − 1.2, − 1.4, and − 1.6 thresholds, respectiveley. Using the RelDiffTh methods, mean (± SD) DICE_PE_ coefficients were 0.67 (0.2), 0.77 (0.14), 0.81 (0.11), and 0.75 (0.16) with the − 30%, − 40%, − 50%, and − 60% thresholds, respectiveley (see Fig. 2).

### Segmentation methods evaluation

#### Comparison of methods

**Fig. 3:**
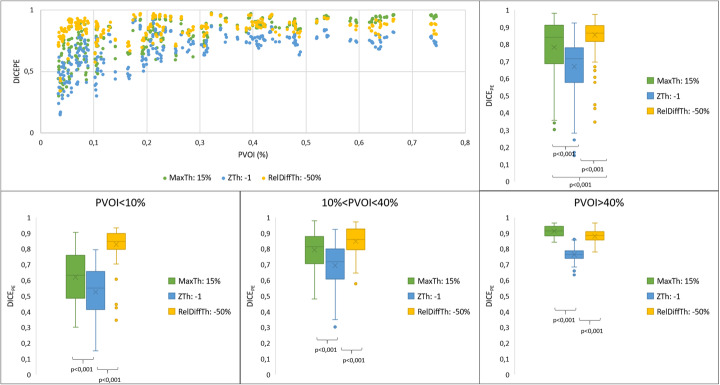
Comparison of the three segmentation methods in terms of DICEPE indices. DICE_PE_ are presented for all PE volumes in the first row and according to the PVOI in the second row. RelDiff method with − 50% threshold shows overall higher DICE_PE_ indices, especially for small and medium PE

Spatial correlation between PE_s_ and PE_ph_ volumes, as assessed by the DICE_PE_ index, is presented in Fig. 3. Mean DICE_PE_ index was significantly higher with the RelDiffTh method (0.85 (0.08)), as compared with the MaxTh method (0.78 (0.16)) and the ZTh method (0.67 (0.15)). Differences between the 3 segmentation methods were more pronouced for small PE (PVOI < 10%), with a mean DICE_PE_ coefficient of 0.62 (0.17), 0.53 (0.15), and 0.83 (0.1) using the MaxTh, ZTh, and RelDiffTh method, respectively. For medium sized PVOI (10% < PVOI< 40%), mean DICE_PE_ coefficients were 0.79 (0.12), 0.7 (0.13), and 0.85 (0.09) for MaxTh, ZTh, and RelDiffTh methods, respectively. For large PVOI (PVOI > 40%), mean DICE_PE_ coefficients were 0.91 (0.04), 0.76 (0.05), and 0.88 (0.04) for MaxTh, ZTh, and RelDiffTh methods, respectively.

**Fig. 4:**
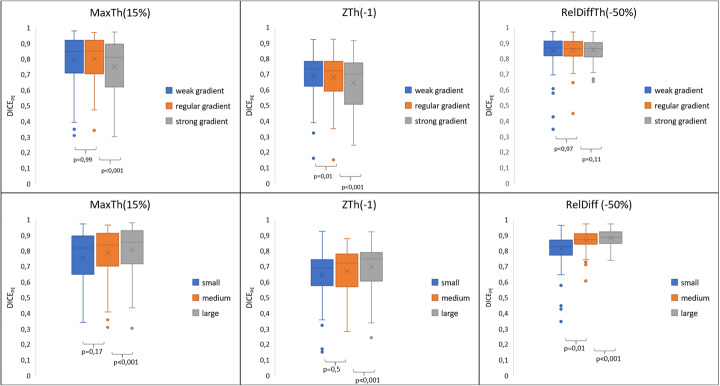
Influences of intensity gradient strenght (first row) and lung volumes (second row) on Dice_PE_ coefficients according to the segmentation methods. Using the RelDiff method with a − 50% threshold, there was no significant influence of the gradient strength and low influence of lung

**Fig. 5:**
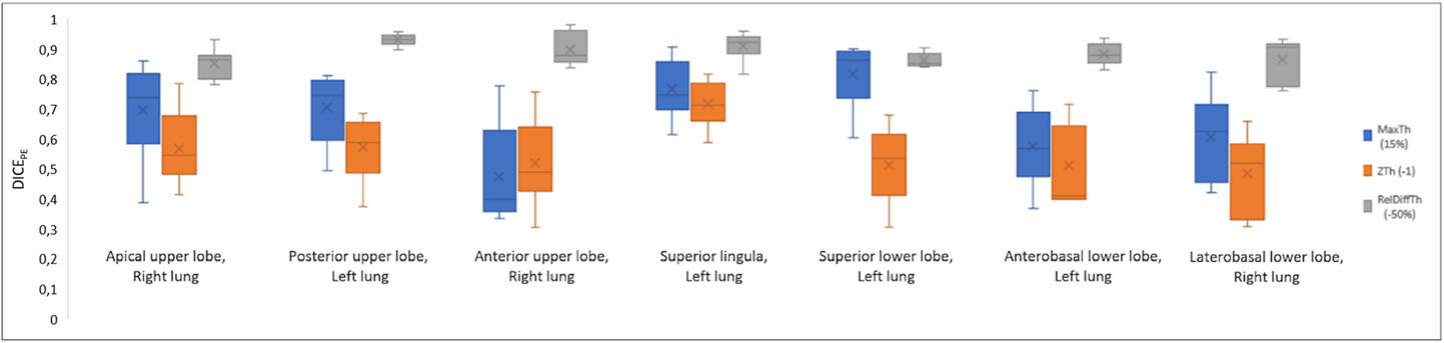
Influence of PE location: high DICE_PE_ were found using the RelDiff method regardless of the location of PE

Figures 4 and 5 showed the impact of the anterior to posterior gradient intensity, lung volume, and PE location on DICE_PE_ distribution according to the segmentation method. The impact of the gradient was much lower for the ReDiffTh method as compared with the MaxTh and ZTh methods (Fig. 4, first row). Using the ReDiffTh method, mean DICE_PE_ was 0.87 (0.08), 0.87 (0.06), and 0.86 (0.06) with the weak, regular, and strong gradient respectively. Lung volumes had an impact on the three segmentation methods, but it was more pronounced for the RelDiffTh methods. Using the ReDiffTh method, mean DICE_PE_ was 0.81 (0.11), 0.87 (0.6), and 0.81 (0.11) with the small, medium, and large lung volumes, respectively.

#### PVOI analysis

**Fig. 6:**
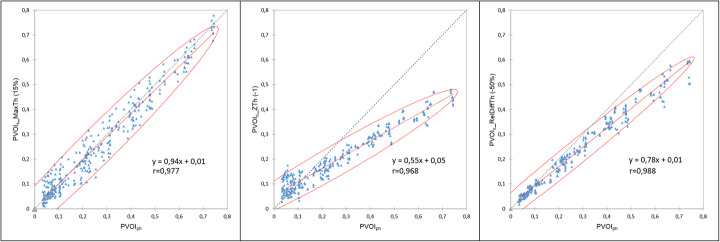
Pearson's correlation between PVOI_ph_ and PVOI_s_ for the three segmentation methods. Person’s coefficient was higher for RelDiff method, but the slope was closer to 1 for MaxTh method

**Fig. 7:**
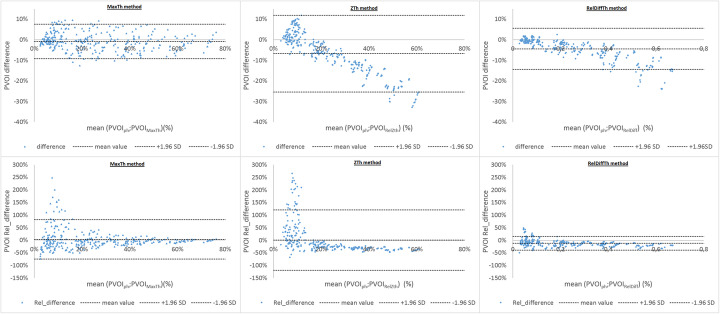
Bland and Altman analysis of the three segmentation methods in terms of absolute difference (first row) and relative difference (second row). RelDiff method showed lower relative differences for medium and small PE

**Fig. 8:**
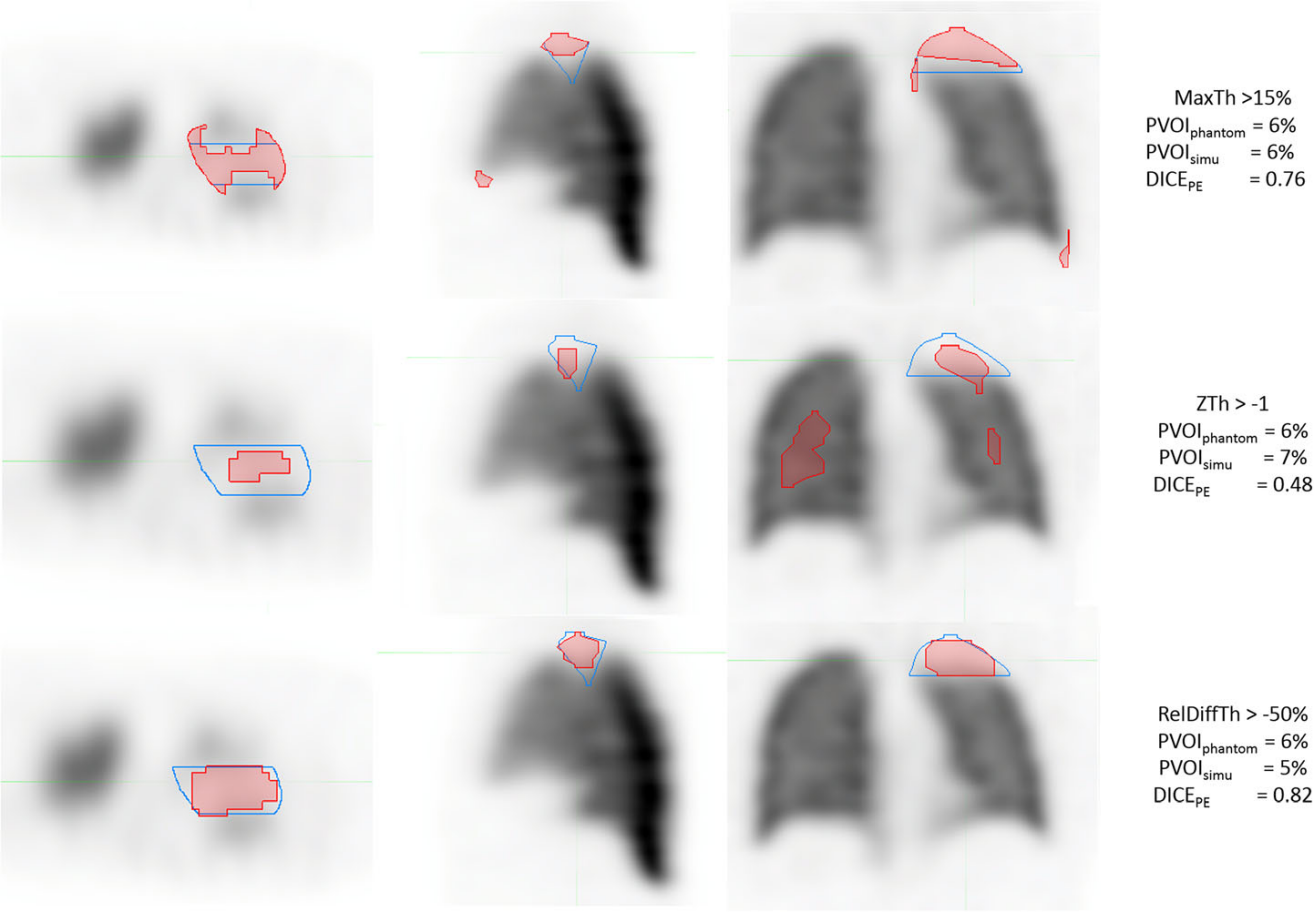
Example of segmentation for a sub-segmental PE (red) using the MaxTh method (first row), the Zth method (second row), and the RelDiffTh method (third row). Ground truth is delineated in blue. The MaxTh and ZTh wrongly delineated areas without PE. RelDiff method showed high accuracy for small PE delineation

**Fig. 9:**
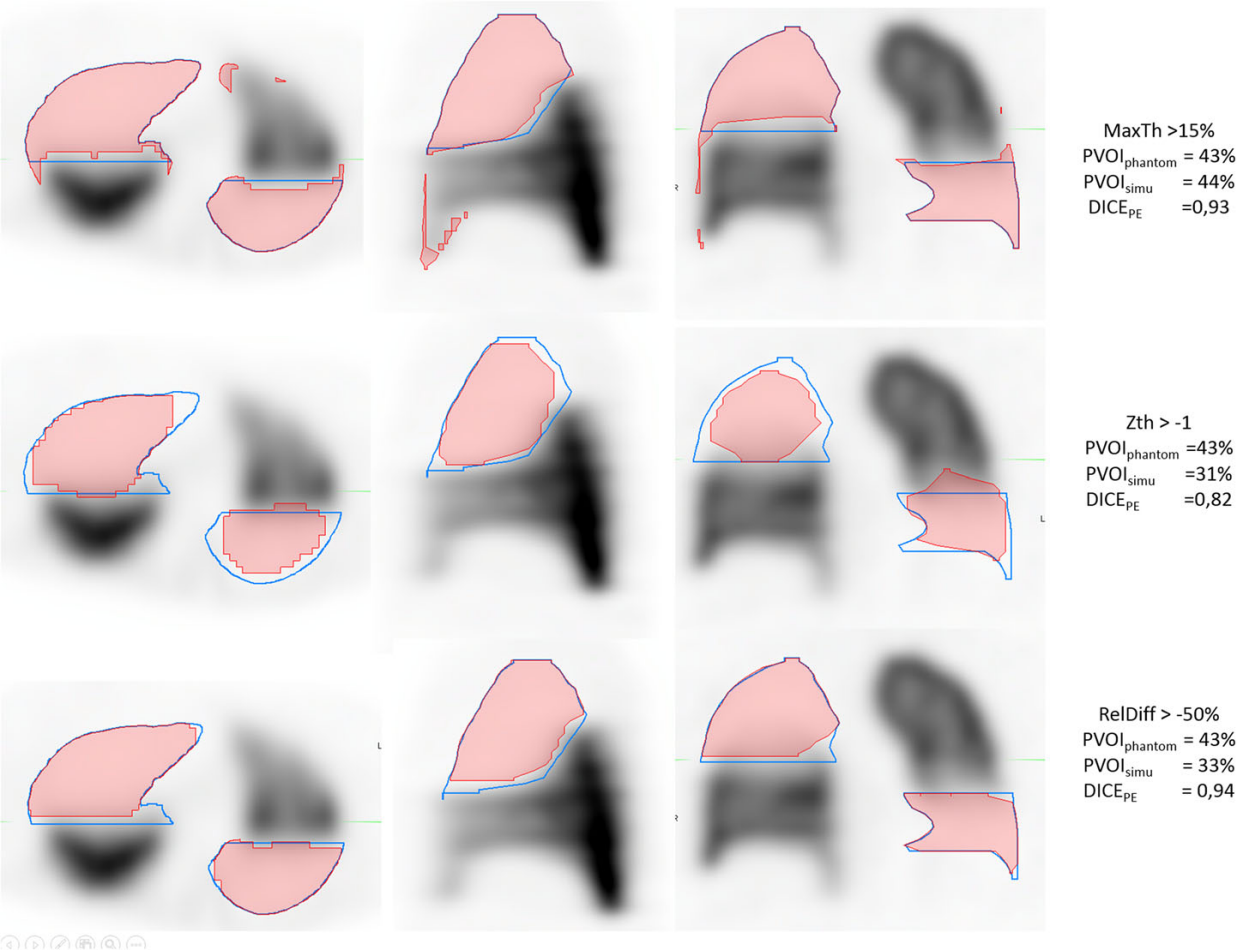
Example of segmentation for a massive PE (red) using the MaxTh method (first row), the Zth method (second row), and the RelDiffTh method (third row). Ground truth is delineated in blue. MaxTh showed good correlation in PVOI assessment, but wrongly delineated areas without PE. RelDiff Method showed an underestimation of PVOI, but a good spatial agreement

The correlation between PVOIs and PVOIph (Fig. 6) was very high for the three segmentation methods (r = 0.977 for MaxTh method, r = 0.968 for ZTh method and r = 0.988 for RelDiffTh method) with a smaller confidence interval for RelDiffTh method. The slope of the regression line was closer to 1 for MaxTh method (0.94 for MaxTh method, r = 0.55 for ZTh method and r = 0.78 for RelDiffTh method). Bland and Altman analysis is presented in Fig. 7 and Table 1. Mean differences and 95% limits of agreement are reported in Table 1. Using the MaxTh method, mean absolute difference in PVOI was − 1%, and limits of agreement were − 9 to 8%. Mean relative difference in PVOI was 3%, and the limits of agreement were − 75 to 81%. Graphical analysis showed that absolute differences were homogenously distributed regardless of the PVOI value, resulting in large relative differences (up to 250 %) for small PVOI indices (< 10%). Using the ZTh method, mean absolute difference in PVOI was − 7%, and limits of agreement were − 25 to 12%. Mean relative difference in PVOI was 0%, and the confidence index was − 120 to 120%. Graphical analysis showed that PVOI were over-estimated for small PE and under estimated for large PE. Using the RelDiffTh method, mean absolute difference in PVOI was − 4%, and limits of agreement were − 14 to 6%. Mean relative difference in PVOI was − 12%, and the confidence index was − 40 to 16%. Graphical analysis show low absolute and relative errors on small and medium PVOI and a trend toward an underestimation of large PVOI. Examples of delineations of a small PE and a massive PE are shown Figs. 8 and 9.

**Table 1:** Bland and Altman analysis: mean absolute and relative differences, with limits of agreement for the three segmentation methods

	MaxTh		ZTh		RelDiffTh	
	Absolute difference	Relative difference	Absolute difference	Relative difference	Absolute difference	Relative difference
Mean difference	− 1%	3%	− 7%	0%	− 4%	− 12%
Limits of agreement	[− 9%; 8%]	[− 75%; 81%]	[- − 25%; 12%]	[− 120%; 120%]	[− 14%; 6%]	[− 40%; 16%]

## Discussion

In this phantom study, we tested and compared three automatic delineation methods of perfusion functional volumes on 396 simulated V/Q SPECT/CT. The best method was a segmentation using a relative difference threshold (− 50%) after coregistration with a parametric normal mean map, which provided good results in terms of spatial correlation and PVOI measurement, regadless of the extent of PE, the intensity of the anterior to posterior gradient, and the whole lung volumes.

The first delineation method consisted in applying to all pixels a fixed intensity threshold defined as a percentage of the maximal pixel value. This has been the most commonly used method for lung segmentation mainly because of its ease of use [21–24]. The overall results showed relatively good spatial agreement with a mean DICE_PE_ = 0.78. Mean relative difference was − 1% (limits of agreement − 75 to 81%). However, while spatial correlation was high for large PE (DICE_PE_ = 0.91 for PVOI_PH_ > 40%), it was lower for small PE (DICE_PE_ = 0.62 for PE < 10%), showing that the method is inaccurate to delineate small PE. Of note is that there was a good correlation in PVOI estimation, although the spatial agreement was poor (low DICE_PE_ value), because segmented non PE volumes were close to non segmented PE volumes (see Fig. 8). Bland and Altman analysis showed a large overestimation of PVOI in a significant proportion of patients with small PE. As illustrated in Fig. 8, this overestimation was mainly due to the delineation of non PE areas around the lungs, especially in their anterior aspect. In a clinical perspective, this represents a major limitation of the method. Indeed, several studies reporting on the pronostic value of PVOI at completion of anticoagulation therapy to predict the risk of PE recurrence or CTEPH, used a PVOI cut-off of 5% or 10% [9]. Accordingly, an overestimation of the PVOI of small PE (e.g., 15–20% while the actual size is < 10%) may have an impact on the therapeutic management of patients. Furthermore, it is worth noting that we did not simulate hot spots, mainly resulting from the accumulation of aerosols in the bronchi, that can make this method based on the intensity of the maximal pixel value totally inaccurate.

The Z-score-based threshold method (ZTh) was conceptually attractive. Indeed, physiological interindividual variabilities of regional lung function is a challenge for automatic delineation of PVOI. Integrating the standard deviation in a pixel by pixel analysis was therefore promising. However, spatial correlation was lower as compared with the 2 others methods, with a mean DICE_PE_ of 0.67, showing that the method is inaccurate to delineate PE. There was a large overestimation of PVOI for small PE, with up to 250% of relative difference, and a systemic under estimation for medium and large PE (around − 50% of relative difference). The over estimation of small PVOI was also due to the delineation of non PE areas, mainly in the center of lungs, as shown in Fig. 8. On the other hand, there was an underestimation of PE volumes in the periphery of the lungs, as shown in Fig. 9. The explanation is provided by the analysis of the normal parametric maps, which show low uptake on mean map and high variability on standard SD maps on the peripheral regions of the lungs (see Fig. 1). This was likely due to free-form registration during the NSDmap creation process, as there is a high variability in patients’ size, and thus in lungs deformation, resulting in the non delineation of PE areas in the periphery of the lungs. Conversely, this issue in the periphery of the lungs led to the choice of a very selective Z-score threshold, which provided the best mean DICE_PE index_, but which resulted in the delineation of non PE areas in the center of lungs.

The relative difference threshold method showed the best results, in particular regarding the spatial correlation and the PVOI measurment for small and medium PE. Mean DICE_PE_ coefficient was the highest (0.85 (0.08)), regardless of the intensity of the anterior to posterior gradient (mean DICE_PE_ = 0.87 (0.08), 0.87 (0.06), and 0.86 (0.06) with the weak, regular, and strong gradient) and the lung volume (mean DICE_PE_ = 0.81 (0.11), 0.87 (0.06), and 0.81 (0.11) with the small, medium, and large lung volumes). However, the PVOI correlation analysis shows a measurement bias, which was also found in the Bland and Altman analysis (relative bias of 12%), but with the smallest confidence interval of the 3 segmentation methods (− 40 to 16%). As discussed above, a key result in a clinical perspective was that PVOI estimation was highly accurate for small and medium size PE. For large PE, there was a trend toward a small underestimation of PVOI, as shown in Fig. 9 . However, the clinical consequences are likely negligible. A likely explanation for this weak underestimation is the non delineation on the edge of the lungs, resulting from the lower spatial resolution of the normal mean map. As mentioned before, hotspots can affect deliniation methods. This method being based on mean intensity value normalization, it should be less affected. Nevertheless, it has to be clinically demonstrated.

There is only few data in the litterature about lung V/Q SPECT delineation in the context of PE. Derlin et al. [21] and Seiffert et al. [24] tested several maximum intensity thresholds and compared them to manual delineation of functional lungs. The best threshold was much higher as compared with our study (40% vs 15%) for Derlin, but closer for Seiffert [24] (18% for left lung and 21% for right lung), illustrating the lake of robustness of the method, especially if there are hot spots. Wang et al. [25–27] proposed three segmentation methods (one threshold based method and two adaptive contouring methods), tested on Monte-Carlo simulations of homogeneous distributions of radioactivity within the lungs. The results, expressed as the ratio of the intersection and the union of the ground truth and the segmented volume, were variable depending on the count rates. The results were up to 97 ± 2 for high count rate, but ranged from 78% ± 23 to 90% ± 9 for low count rates, although the measurement was performed on the functioning lungs (and not on PE) and on simulated data without the intensity gradient. Cheimariotis et al. [28] proposed an active shape segmentation method and compared it with manual delineation. Although DICE indices were calculated on the functional lungs (i.e., not on PE volumes), which inherently improve the correlation of segmented lung volumes, the Dice coefficients were lower than in our study (DICE = 0.82 and 0.83 for left and right lungs respectively).

Our study has some limitations. First, we used a static plantom and we not simulated respiratory motion. However, based on the analysis of 73 normal coregistrated cases database [14], the impact of respiratory motion on the uptake variability in the basis areas was very low, especially as compared with the anterior to posterior gradient. Second, we only focused the analysis on perfusion images while the hallmark of acute PE with V/Q scintigraphy is the mismatched perfusion defect, i.e., areas with absent perfusion but preserved ventilation. There are many pulmonary conditions, such as chronic obstructive pulmonary disease or pneumonia, which can be the source of matched defects present on both perfusion and ventilation. On the other hand, the Meyer score [11] as long as recent studies that demonstrated the predictive value of the PVOI on PE recurrence [9, 10] only used perfusion images for PVOI quantification. Third, the DICE coefficients used in the calibration process, calculated on PE volumes, were relatively low and showed a large range, especially for the MaxTh and ZTh methods, which may question the reliability of the selected threshold and limit its generalizability. Of note however is that the DICE coefficients were much higher for the relative difference method. Fourth,while Monte Carlo simulation offers many advantages, including the reliability of the reference standard and the possibility to assess multiple methods and clinical scenario, it remains a phantom study. In particular, perfusion defects were artificially delineated and would not account for real patient variability. Thus, it is not possible to extrapolate our results to real V/Q SPECT/CT scans. Clinical studies are now warranted to assess whether this segmentation method can be used in daily practice to compute the PVOI.

## Conclusion

In this phantom study, a delineation method based on a relative difference parametric map provided a good estimation of the PVOI, regardless of the extent of PE, the intensity of the anterior to posterior gradient, and the whole lung volumes. As compared with manual segmentation method or segment counting method, this automated delineation method may provide a fast, accurate, and reproducible PVOI quantification. Clinical studies are now required to assess whether this segmentation method can be used in daily practice to compute the PVOI.

## Supplementary Information

**Additional file 1.**

## Data Availability

The datasets used and analyzed during the current study are available from the corresponding author on reasonable request.

## Abbreviations

PE: Pulmonary embolism
V/Q: Ventilation/perfusion scan
SPECT: Single photon emission computed tomography
CT: Computed tomography
PVOI: Pulmonary vascular obstruction index
MaxTh: Relative to maximum intensity threshold method
ZTh: Z-score threshold method
RelDiffTh: Relative difference threshold method
CTEPH: Chronic thromboebolic pulmonary hypertension
SPM: Statistical parametric mapping
MELP: Medium energy low penetration
WLv_PH_: Phantom’s whole lungs volume
WLv_S_: Simulation’s whole lungs volume
FLv_PH_: Phantom’s functional lungs volume
FLv_s_: Simulation’s functional lungs volume
PEv_PH_: Phantom’s pulmonary embolism volume
PEv_S_: Simulation’s pulmonary embolism volume
PVOI_PH_: Phantom’s pulmonary vascular obtruction index
PVOI_s_: Simulation’s pulmonary vascular obtruction index
DICE_PE_: DICE index between PEv_PH_ and PEv_S_
