# Supplementary material for: Automatic delineation and quantification of pulmonary vascular obstruction index in patients with pulmonary embolism using Perfusion SPECT-CT: a simulation study
Source: EJNMMI Phys. 2021 Jul 5;8:49. doi: 10.1186/s40658-021-00396-1 (PMC8257882; doi:10.1186/s40658-021-00396-1)
Supplement: Supplementary file 1 — Additional file 1. [file 40658_2021_396_MOESM1_ESM.pdf]

PE scenarii definition

Code	Anatomical definition
L1	Right upper lobe (RUL)
L2	Right middle lobe (RML)
L3	Right lower lobe (RLL)
L4	Left upper lobe (LUL)
L5	Left lower lobe (LLL)
L6	LLL + RUL
L7	RLL + RML
S5 1	Right S1 (RS1)
S5 2	Left S2 (LS2)
S5 3	RS3
S5 4	LS4
S5 5	RS5
S5 6	LS6
S5 7	RS7
S5 8	LS8
S5 9	RS9
S5 10	LS10
S10 1	RS2+LS3
S10 2	RS6+LS9
S10 3	RS4+LS5
S20 1	LS2+RS1+RS10+LS4
S20 2	RS5+RS6+LS10+LS5
S20 3	LS1+LS6+RS7+RS3
S30 1	RS1+RS4+RS6+LS3+LS5+LS10

S30 2	RS3+RS5+RS8+LS1+LS4+LS7
S30 3	RS2+RS7+RS8+LS4+LS6+LS8
S40 1	RS2+RS4+RS5+RS9+RS10+LS6+LS7
S40 2	RS3+RS8+RS9+LS4+LS5+LS9+LS10+LS3
S40 3	RS4+RS5+RS6+RS8+LS1+LS3+LS6+LS8
S50 1	RS1+RS2+RS3+RS6+RS10+LS4+LS5+LS8+LS9+LS10
S50 2	RS4+RS5+RS2+RS9+RS8+LS1+LS3+LS7+LS8+LS10
S50 3	RS1+RS4+RS6+RS10+RS8+LS3+LS5+LS8+LS7+LS10
s60 1	RS1+RS3+RS5+RS6+RS7+RS9+LS1+LS2+LS3+LS4+LS9+LS10
s60 2	RS2+RS4+RS5+RS8+RS9+RS10+LS3+LS5+LS6+LS7+LS8+LS10
s70 1	RS1+RS3+RS4+RS5+RS7+RS8+RS9+LS1+LS2+LS4+LS5+LS7+LS8+LS9
s70 2	RS2+RS3+RS5+RS6+RS8+RS9+RS10+LS1+LS2+LS3+LS6+LS7+LS8+LS10
SS5 1	subRS6+subRS5
SS5 2	subRS5+subLS1
SS10 1	subRS1+subRS10+subRS6+subLS1
SS10 2	subRS9+subRS1+subLS8+subRS5
SS15 1	subRS1+subRS2+subLS8+subLS10+subRS5+subLS1
SS15 2	subRS6LS5+subRS2+subLS1+subLS5+subLS10
ss20 1	subRS2+subRS3+subRS5+subRS9+subLS2+subLS4+subLS6+subLS8
ss20 2	subRS1+subRS4+subRS6+subRS8+subLS1+subLS3+subLS7+subLS9
